# A Patient With Encephalomyeloradiculoneuropathy Exhibiting a Relapsing–Remitting Clinical Course: Correlation of Serum and Cerebrospinal Fluid Anti-Neutral Glycosphingolipids Antibodies With Clinical Relapse

**DOI:** 10.3389/fneur.2018.00206

**Published:** 2018-04-04

**Authors:** Hitoki Nanaura, Hiroshi Kataoka, Sayuri Shima, Naoki Iwasa, Nobuyuki Eura, Kazuma Sugie, Tatsuro Mutoh, Satoshi Ueno

**Affiliations:** ^1^Department of Neurology, Nara Medical University, Kashihara, Japan; ^2^Department of Neurology, Fujita Health University School of Medicine, Toyoake, Japan

**Keywords:** encephalomyeloradiculoneuropathy, glycosphingolipids, lactosylceramide, antibody, encephalopathy, neuropathy, myelopathy

## Abstract

Several patients who had a progressive clinical course involving both the central and peripheral nervous systems have been reported, but the diagnostic marker has been remained uncertain. More recently, such patients were reported to have namely “encephalomyeloradiculoneuropathy (EMRN)” associated with anti-neutral glycosphingolipid (GSL) antibodies. These antibodies were reported to disappear from the serum in the recovery phase, but whether this finding applies to the cerebrospinal fluid (CSF) remains uncertain. We describe a 67-year-old man with EMRN in whom we measured anti-neutral GSL antibodies in serial serum and CSF samples. During the disease course, the optical densities of the positive band against the background intensity ratio (–<0.3; ±≥0.3 to <0.6; +≥0.6 to <1.0; 2+≥1.0 to <2.0; 3 +≥2.0) for serum and CSF anti-lactosylceramide (LacCer) antibodies were found to be as follows: 2+ and 1+ at the first admission, ± and − when the consciousness level improved after immunotherapy, − and 1+ at clinical relapse, and ± and − when the consciousness level improved after immunotherapy. This is the first time to document that clinical relapse occurred in EMRN, and at this time the negative anti-LacCer antibodies in CSF after the first course of immunotherapy turned positive, but this was not seen in serum samples.

## Background

In 1968, Blennow et al. were the first to describe three patients who had a progressive clinical course involving both the central (CNS) and peripheral nervous systems (PNS) (1), and similar cases have been reported subsequently (2). However, a diagnostic clinical biomarker for this condition remains to be determined. In 2013, Kawamura et al. found that anti-neurofascin-155 antibodies sometimes turn positive in such patients (3). More recently, four patients who had CNS and PNS disorders were reported to have namely “encephalomyeloradiculoneuropathy (EMRN)” associated with anti-neutral GSL antibodies, especially anti-lactosylceramide (LacCer) antibodies (4). Neutral glycosphingolipids (GSLs) are distributed in the cell membranes of myelin-forming cells, such as oligodendrocytes and Schwann cells as well as neurons and immune cells. These antibodies were reported to disappear from the serum in the recovery phase (4), but whether this finding applies to the cerebrospinal fluid (CSF) remains uncertain. We describe a patient with EMRN in whom we measured the anti-LacCer antibodies in serial serum and CSF samples.

## Case Report

A 67-year-old man received oral prednisolone (from 15 to 20 mg/day for 1 year) because of remitting seronegative symmetrical synovitis with pitting edema syndrome. He also had a history of cerebral infarction at 62 years of age, and mild right residual hemiparesis was evident, but an assistant was not needed to perform activities of daily living. Difficulty in walking or standing developed over a period of 4 weeks, and he was admitted to our hospital. At this time, he was receiving oral prednisolone (15 mg/day). The score on the Glasgow Coma Scale (GCS) was 14 (E4V4M6). Severe motor weakness and moderate superficial sensations in all four limbs, especially the lower limbs, were evident, and he could not stand by himself. Deep-tendon reflexes of the four limbs were increased. Babinski’s sign was negative. Ankle clonus was present bilaterally. Urinary frequency was decreased to a few times per day. Mild bilateral ocular abduction with subjective diplopia was present.

**Figure 1:**
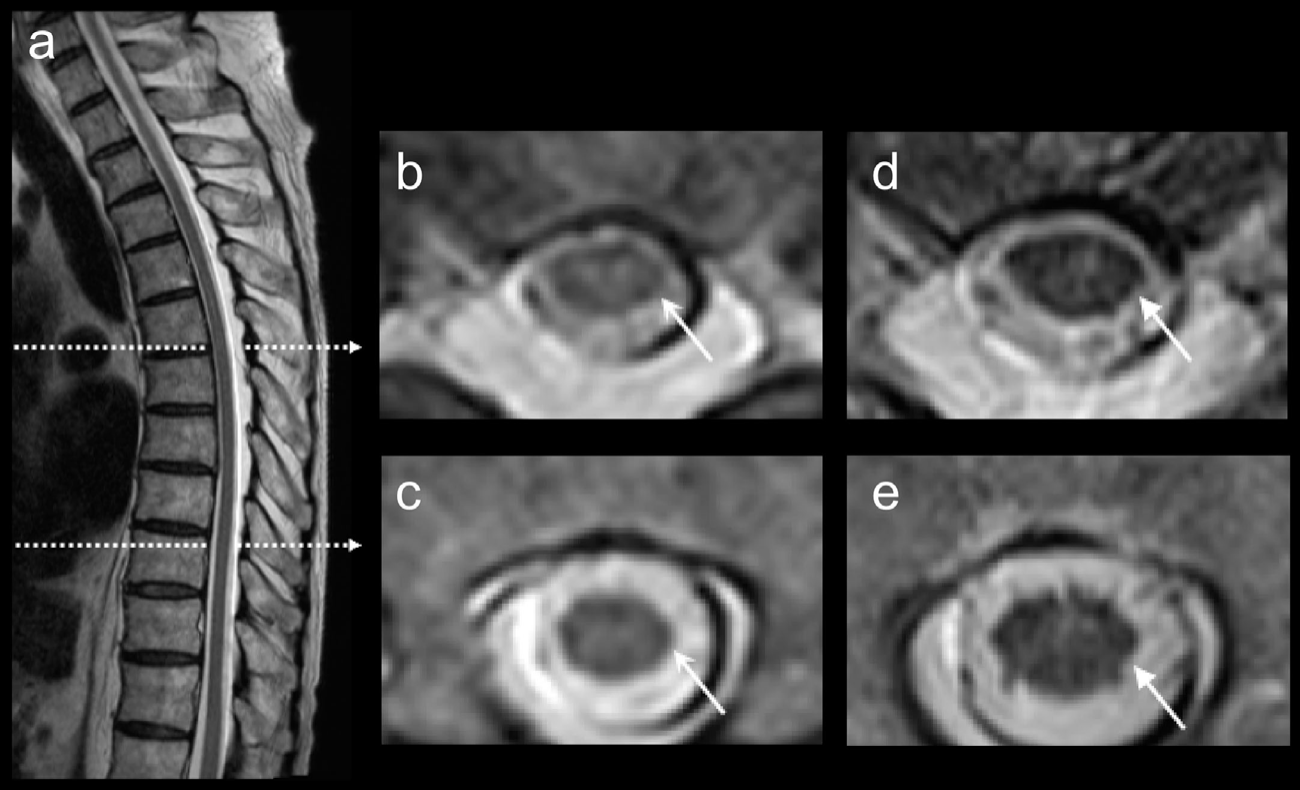
Thoracic magnetic resonance imaging (MRI). T2-weighted thoracic MRI showed abnormal high signals in the spinal cord **(A–C)**. After 5 months, the high signals were diminished on the follow-up thoracic MRI **(D,E)**.

Laboratory findings were normal expect for glucose (80 mg/dl) and hemoglobin A1c (6.6%) levels. Spinal magnetic resonance imaging (MRI) showed abnormal high-intensity signals in the thoracic spinal cord (Figure 1). The cranial MRI was normal except for old cerebral infarction in the left tempo-parietal lobe. CSF analysis showed a normal cell count and an increased protein concentration (101 mg/dl) and IgG index (0.79). Oligoclonal bands and myelin basic protein were absent. Polymerase chain reaction analyses for herpes simplex virus, cytomegalovirus, and human herpes virus 6 were negative, and CSF antibodies to varicella-zoster virus and human T-cell leukemia virus type 1 were not elevated. Electroencephalography (EEG) showed focal delta activity in both frontal regions. Nerve conduction studies of the four limbs demonstrated reduced amplitudes of compound muscle action potentials (CMAPs) in the median and ulnar nerves (right and left: 3.4 and 7.3 mV, respectively), the right ulnar nerve (7.6 mV), and the tibial nerve (0.63 mV), and CMAPs of the left tibial nerve and both peroneal nerves were not evoked. The conduction velocities were reduced in the left ulnar nerve (46.3 m/s). Distal motor latencies of the right median (4.0 ms) and tibial nerves (5.5 ms) were mildly prolonged. Sensory nerve action potential was not evoked in the right sural nerve. F-wave latencies of the right median nerve (29.0 ms) were mildly prolonged, and F-waves were absent in the left ulnar and tibial nerves and both peroneal nerves. Motor-evoked potential on magnetic stimulation showed a prolonged central motor conduction time (35.1 ms >18.5 ± 4.0 ms). Sural nerve biopsy showed decreased myelin fibers with no fibrinoid necrosis, inflammatory cell infiltration, or granuloma. Macroscopic examination showed segmental demyelination. Malignancy including myeloma or lymphoma was absent on whole-body computed tomography, gastrointestinal endoscopy, gallium scintigraphy, bone marrow biopsy, and random skin biopsy.

**Figure 2:**
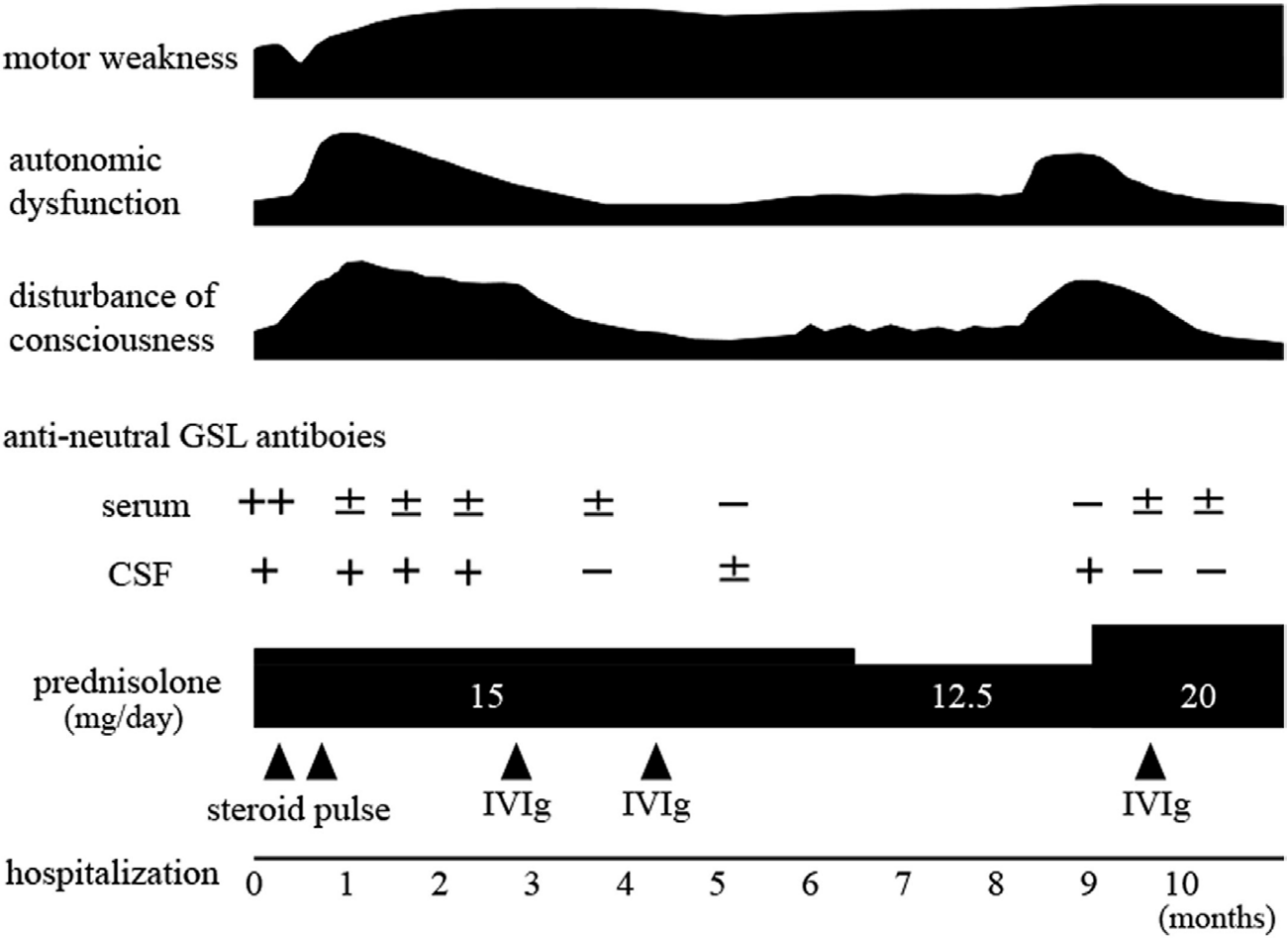
Clinical course and serum and cerebrospinal fluid anti-lactosylceramide antibodies.

The clinical course is shown in Figure 2. The patient received steroid pulse therapy (1 g/day, 3 days) two times. The motor weakness further progressed, and 1 month after admission, the GCS score fell to 8 (E2V2M4). Subsequently, postural hypotension or persistent tachycardia (heart rate: 120–160/min) was evident, and urinary dysfunction developed, requiring a balloon catheter. Postural hypotension (from systolic 100 mmHg in supine position to 60 mmHg in sitting position) also occurred. The patient was given intravenous immunoglobulin (IVIg, 400 mg/kg/day, 5 days, two times). After the first treatment with IVIg, the GCS score increased to 14 (E4V4M6), and the focal delta activity on EEGs disappeared. The motor weakness slightly improved. The severity of postural hypotension (from systolic 108 mmHg in supine position to 106 mmHg in standing position) was diminished. The frequency of tachycardia also decreased, and the urinary balloon catheter was withdrawn. Six months after admission, he was transferred to a rehabilitation hospital. Nine months after the first admission, the level of consciousness fell again, and he was readmitted to our hospital. The GCS score was 11 (E3V3M5) with complete paraplegia, urinary dysfunction requiring use of a catheter, and postural hypotension. The frontal delta activity reoccurred on EEGs. The dose of oral prednisolone was increased to 20 mg/day, and subsequently a third course of IVIg (IVIg, 400 mg/kg/day, 5 days) was initiated. The GCS score increased to 14 (E4V4M6). The severity of both urinary dysfunction and postural hypotension decreased. Ten months after the first admission, he could move using a wheelchair unaided. He was transferred to the rehabilitation hospital because of motor weakness. The protein concentration and IgG index in CSF did not decrease during this clinical course.

### Autoantibody Measurements

**Figure 3:**
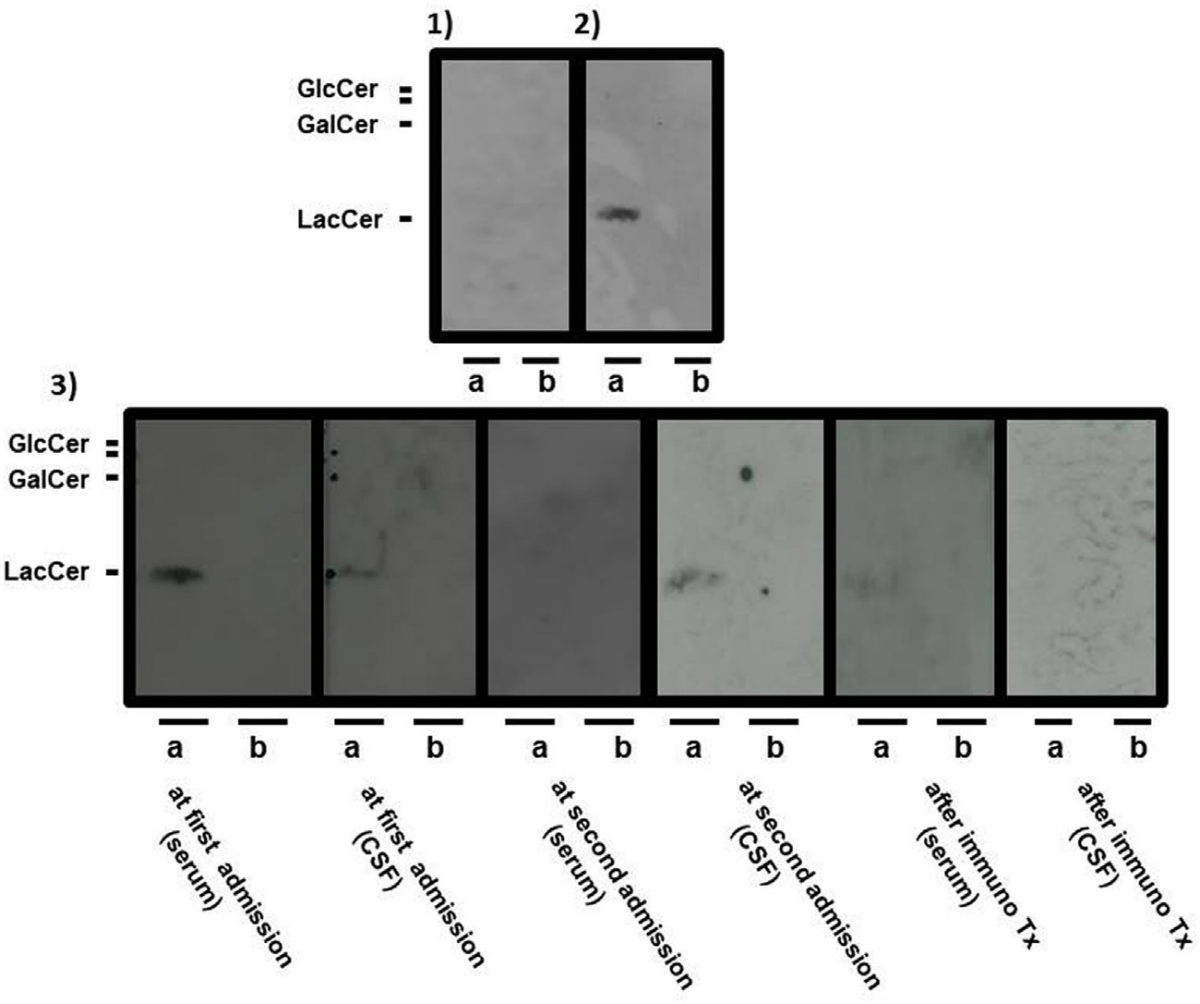
Serial Far-Eastern blot analyses. Neutral glycosphingolipids (GSLs), such as lactosylceramide, glucosylceramide, and galactosylceramide (GalCer) were applied onto thin-layer chromatography plates (TLC plates) to obtain the respective pure neutral GSLs, and they were developed as described previously (4). The separated neutral GSLs were transferred to a regular polyvinylidene difluoride membrane (PVDF membrane) with a thermal blotter (ATTO, Tokyo, Japan). The positive band(s) were detected with a chemiluminescence detection system (4). The separated GSLs were probed with serum from a healthy volunteer [(1): negative control] and with serum from a patient with encephalomyeloradiculoneuropathy [(2): positive control]. Panel (3) shows the separated GSLs from the patient at the following multiple time-points: serum at the first admission, cerebrospinal fluid (CSF) at the first admission, serum at the second admission, CSF at the second admission, serum after final immunotherapy, and CSF after final immunotherapy. Positive bands were detected with serum and CSF from the patient at the first admission and CSF from the patient at the second admission. A positive band was weakly detected with serum from the patient after final immunotherapy. Sera and CSF are usually 1,000- and 5-times diluted with the antibody reaction buffer, respectively. Lactosylceramide and glucosylceramide were applied to (a) lane, whereas GalCer was applied to each (b) lane of the TLC plates.

Anti-neutral GSL antibodies (IgG fraction) in serial serum and CSF samples were measured by Far-Eastern blot analyses as described previously (4). For quantification, we subjected all membranes to image analysis using NIH Image software and calculated the ratio of signal intensity to background intensity as described previously (4). Sera and CSF were usually 1,000- and 5-times diluted with buffer, respectively. During the disease course, the optical density ratios of serum and CSF anti-LacCer antibodies were as follows: 2+ and 1+ at the first admission, ± and 1+ when the consciousness level decreased despite steroid pulse therapy, ± and − when the consciousness level improved after the first IVIg treatment, − and 1+ at clinical relapse, and ± and − when the consciousness level improved after increasing the dose of prednisolone and administering the third IVIg treatment (optical density ratio: −<0.3; ±≥0.3 to <0.6; +≥0.6 to <1.0; 2 +≥1.0 to <2.0; 3 +≥2.0) (Figure 3). Anti-galactosylceramide (GalCer), glucosylceramide and ceramide antibodies were negative. Anti-GM1, GM2, GM3, GD1a, GD1b, GT1b, and GQ1b antibodies were negative at the first admission; only anti-GD3 IgM antibody was positive. Anti-sulfated glucuronyl paragloboside (SGPG), anti-neurofascin 155, aquaporin-4, and myelin oligodendrocyte glycoprotein (MOG) antibodies were all negative. Anti-amphiphysin, CV2, PNMA2 (Ma2/Ta), Ri, Yo, Hu, recoverin, SOX1, titin, zic4, GAD65, Tr (DNER) antibodies, anti-nuclear antibody, anti-SS-A antibody, anti-SS-B antibody, PR3-ANCA, and MPO-ANCA were also absent.

Written informed consent for clinical cure and care, including all treatments and the measurement of all antibodies, was obtained from both the patient and his family. Written informed consent was also obtained from both the patient and his family for publication of this case report.

## Discussion

Similar to four previously documented patients with anti-neutral GSL antibodies (4), our patient had subacute motor weakness, decreased consciousness, elevated CSF protein levels, spinal disorder, peripheral axonal and demyelinating polyneuropathy, and autonomic dysfunction. As for MRI findings, the increased signals in the spinal cord were diminished in the recovery phase as reported previously (4). Neutral GSL exists in the cell membranes of myelin-forming cells, but our patient showed primary axonal neuropathy on nerve conduction studies. Some patients with acute inflammatory demyelinating polyneuropathy, such as axonal Guillain–Barré syndrome, show decreased motor or sensory amplitudes, or both, on nerve conduction studies (5), and IgG, and complements were deposited on the nodal axolemma of rat sciatic nerves after the injection of anti-ganglioside antibodies (6). The reason why our case exhibited mainly axonopathy mixed with demyelination to a lesser extent is not known at present because patients who had PNS disorder associated with anti-LacCer antibodies have not been reported previously. Two of four patients with this antibody (4) showed primary axonal neuropathy, and the other two patients had the axonal neuropathy in addition to demyelinating neuropathy. Our patient showed segmental demyelination on the macroscopic examination of a sural nerve biopsy specimen.

Previous patients who had CNS and PNS disorder associated with anti-neutral GSL antibodies did not have a relapse probably because of the short-term follow-up periods (4). Our patient is the first time to document the clinical relapse of EMRN, and at relapse the negative anti-LacCer antibodies in CSF after the first course of immunotherapy turned positive, but this was not seen in serum samples. Similar findings have been reported in patients with anti-*N*-methyl-d-aspartate receptor encephalitis, suggesting that CSF antibody testing correlates better with relapses than does serum antibody testing (7). The reason why anti-LacCer antibodies in the CSF became positive despite the absence of serum antibodies at clinical relapse might be explained by the properties of the antibodies. In our patient, the protein concentration and IgG index in CSF did not decrease before clinical relapse, suggesting that plasma cell or immunoglobulin production continued at very low levels in the CSF, whereas in all previously reported cases of EMRN, protein concentrations and IgG indexes in CSF normalized during the recovery phase of the disease (4). The reactivation of plasma cells in the CSF might reproduce anti-LacCer antibodies. Another possible reason might be explained by the use of different dilutions for detecting serum or CSF antibodies. The absence of oligoclonal bands in CSF might also be explained by the very low levels of CSF IgG because the correlation between the number of oligoclonal bands and CSF IgG is not linear even if the samples are obtained from patients with multiple sclerosis (8).

How anti-LacCer antibodies act on the antigen on myelin-forming cells is uncertain. As for acute inflammatory demyelinating polyneuropathy, the IgG1 or IgG3 subclass of anti-ganglioside antibodies is generally associated with T-cell-dependent responses to antigens on the motor nerves together with complement activation (9). In CNS and PNS disorders associated with neurofascin-155 antibodies, IgG4 subclass antibodies act on their antigenic target *via* direct interference (10). The subclass of the anti-LacCer is the IgG1. Anti-GalCer antibodies, one type of anti-neutral GSL antibodies, cause demyelinating neuropathy in rabbits (11). Anti-GalCer antibodies obtained from rabbits bind to GalCer in both central and peripheral myelin in sections of rat optic and sciatic nerves (12). Different anti-LacCer monoclonal antibodies, T5A7 and Huly-m13, in mouse recognize different sites in the LacCer domain on the surface of mouse blood cells, suggesting that the anti-LacCer antibodies might interact with antigen (13).

The severity of disease was improved by immunotherapy, especially the level of consciousness. In the three previous patients (4), treatments with high-dose immunoglobulins or steroids resolved the neurological findings or radiologic abnormalities. In our patient, the motor weakness was unchanged, suggesting that second-line treatments such as immunosuppressants may be required. When clinicians encounter a patient with both CNS and PNS involvement, not only serum samples, but also CSF samples should undergo anti-neutral GSL antibody testing, especially when clinical relapse has occurred.

## Ethics Statement

No investigations or interventions were performed outside of routine clinical care for this patient. As this is a case report, without experimental intervention into routine care, no formal research ethics approval was required. Written, fully informed consent was obtained from the patient. This case study describes routine clinical care provided for a patient only.

## Author Contributions

HN and HK were responsible for the overall study design, and wrote the manuscript. HN, TM, SS, HK, NI, NE, and KS contributed to acquisition of the data. HN, HK, and TM contributed to analysis and interpretation of the data. HK, TM, and SU contributed to drafting and critical revision of part of the submitted materials.

## Conflict of Interest Statement

The authors declare that the research was conducted in the absence of any commercial or financial relationships that could be construed as a potential conflict of interest. The reviewer RY and handling editor declared their shared affiliation.
